# Accelerated Corneal Collagen Cross-Linking in Pediatric Patients: Two-Year Follow-Up Results

**DOI:** 10.1155/2014/894095

**Published:** 2014-09-11

**Authors:** Rohit Shetty, Harsha Nagaraja, Chaitra Jayadev, Natasha Kishore Pahuja, Mathew Kurian Kummelil, Rudy M. M. A. Nuijts

**Affiliations:** ^1^Narayana Nethralaya Eye Hospital, 121/C Chord Road, 1 “R” Block, Rajajinagar, Bangalore, Karnataka 560 010, India; ^2^University Eye Clinic Maastricht, Maastricht University Medical Center, Maastricht, The Netherlands

## Abstract

*Purpose.* To evaluate the effectiveness and safety of accelerated corneal collagen cross-linking (ACXL) in patients below 14 years of age with progressive keratoconus.* Materials and Methods*. Thirty eyes of 18 patients with established progressive keratoconus underwent preoperative and postoperative visual acuity assessment, topography, and specular microscopy prior to ACXL and were followed up for 24 months.* Results.* Mean age of the patients was 12.7 years with ten males and eight females. There was an improvement in the mean postoperative uncorrected distant visual acuity (from 0.76 ± 0.26 to 0.61 ± 0.25; *P* = 0.005), mean corrected distant visual acuity (from 0.24 ± 0.19 to 0.12 ± 0.12; *P* < 0.001), mean spherical refraction (from −3.04 DS ± 3.60 to −2.38 DS ± 3.37; *P* = 0.28), mean cylinder (from −3.63 DC ± 1.82 to −2.80 DC ± 1.48; *P* = 0.008), and spherical equivalent (from −4.70 D ± 3.86 to −3.75 D ± 3.49; *P* = 0.15). Three eyes of two patients with vernal keratoconjunctivitis (VKC) showed progression. There were no intra- or postoperative complications.* Conclusion.* In pediatric patients ACXL is an effective and safe procedure for the management of keratoconus. Optimal management of VKC is important to arrest the progression of keratoconus.

## 1. Introduction

Keratoconus is characterized by progressive corneal protrusion and thinning, leading to irregular astigmatism and impairment in visual function, secondary to changes in the structure and organization of corneal collagen [1, 2]. The ectasia progresses at a variable rate but may be more rapid in pediatric patients afflicted with vernal keratoconjunctivitis (VKC) [3]. Reeves et al. found that keratoconus progression was more frequent and faster in patients under 18 years of age, with a seven-fold higher risk of requiring corneal transplantation [4]. There have been some studies that have used corneal collagen cross-linking (CXL) in the management of young patients with progressive keratoconus and found it to be effective [5]. Corneal collagen cross-linking causes photopolymerization of the stromal collagen fibers by using the combined action of a photosensitizing substance (riboflavin or vitamin B2) and ultraviolet- (UV-) A irradiation. This results in corneal stiffening due to an increase in the number of intrafibrillar and interfibrillar covalent bonds with heightened corneal collagen resistance against enzymatic degradation [6, 7]. A shorter duration of treatment may offer some advantage to the pediatric age group. Hence we undertook a pilot study to analyze the safety and effectiveness of “accelerated” collagen cross-linking (ACXL) in patients under 14 years of age.

## 2. Materials and Methods

This was a prospective interventional study of 30 eyes of 18 patients. The inclusion criteria for the study were eyes with progressive keratoconus documented by serial topography for at least six months, corneal thickness >400 microns at the thinnest location, and children in the age group of 11–14 years. An increase in the steep K-value by more than 1.0 to 1.5 D and a corresponding change (form >1.0 to 1.5 D) in the subjective refraction in the last six months or a 5% or more decrease in the thinnest pachymetry in the preceding six months was defined as “progression.”

Eyes with corneal thickness <400 microns at the thinnest point, concurrent corneal infections, central or paracentral scarring, and those who had a history of herpetic keratitis were excluded.

Written informed consent was obtained from parents of all patients undergoing the procedure, and the study protocol was approved by the hospital's ethics committee and was performed according to the tenets of the Declaration of Helsinki. All patients underwent a detailed ophthalmic examination including assessment of the uncorrected distant visual acuity (UDVA) and corrected distant visual acuity (CDVA), subjective acceptance, slit lamp, specular microscopy, and dilated fundus examination. Both UDVA and CDVA were recorded using Snellen's chart and later converted to logMAR values. All patients underwent corneal topography using the Scheimpflug camera Pentacam (Oculus, Wetzlar, Germany). Keratometric values (K1 and K2) and minimum pachymetry values were derived from the Pentacam and the pachymetry was confirmed with an ultrasound pachymetry. All patients underwent the above tests at baseline and at all subsequent visits.

### 2.1. Surgical Technique

Corneal collagen cross-linking was performed under sterile conditions in the operating room. Topical proparacaine hydrochloride 0.5% eye drops were instilled preoperatively. The central 8 mm of the corneal epithelium was removed using an epithelial scraper. Riboflavin 0.1% solution (10 mg riboflavin-5-phosphate in 10 mL dextran-T-500 20% solution) was applied as a photosensitizer every 2 minutes for 30 minutes. After confirming permeation of riboflavin through the cornea using a PSL Portable Slit Lamp (Reichert, Depew, NY), UV-A irradiation of 9 mW/cm^2^ with a wavelength of 365 nm was initiated using the Avedro KXL system (Waltham, MA) for 10 minutes. During irradiation, drops of riboflavin solution were reapplied to the cornea every two minutes to sustain the necessary concentration and to prevent desiccation of the cornea. A 9 mm sized beam was used with care taken to avoid any damage to the limbus by using a limbal guard made of polymethyl methacrylate. A silicone hydrogel bandage contact lens (Pure Vision, Bausch and Lomb) was applied following the treatment and was removed on the 3rd postoperative day or once the epithelium healed.

Postoperative treatment included prednisolone acetate 1% eye drops in tapering dosage for three weeks, moxifloxacin hydrochloride 0.5% eye drops for one week, nepafenac 0.1% eye drops three times a day for three days, and topical artificial tears supplements for three months. Patients with associated VKC were treated with topical antiallergic medication and cyclosporine as prophylaxis to prevent acute exacerbations.

For residual refractive errors three months after ACXL, patients were prescribed contact lenses (rigid gas permeable, Rose-K, or hybrid lenses). Patients who had allergic eye disease or were intolerant to contact lenses were given spectacle correction.

### 2.2. Statistical Analyses

The raw data was entered on excel sheets (Microsoft Corp.) and imported to the Statistical Package for Social Sciences (SPSS Inc., Chicago, IL, version 17.0) for analysis. We used both eyes of patients when eligible. As the outcomes are likely to be correlated between the two eyes of a patient, we used generalized estimating equations to adjust for the same during all statistical comparisons. The significance level was set at <0.05.

## 3. Results

Thirty eyes of 18 patients were included in the study with ten males and eight females; mean age of the patients was 12.7 years (range: 11–14 years). Seventeen eyes (56.7%) of 10 patients (55.5%) had an associated vernal keratoconjunctivitis (VKC). The mean pachymetry as measured by the Pentacam was 453.14 microns (range: 432–510 microns).

Table 1 shows the changes in UDVA, CDVA, spherical equivalent (SE), K1 (flat K), and K2 (steep K) preoperatively and 24 months postoperatively.

### 3.1. UDVA and CDVA (in LogMAR)

The mean preoperative UDVA and CDVA were 0.76 (±0.26) and 0.24 (±0.19), respectively. At the end of two years, there was a statistically significant change in the mean UDVA (0.61 ± 0.25; *P* < 0.001) and mean CDVA (0.12 ± 0.12; *P* < 0.001).

### 3.2. Spherical, Cylinder, and Spherical Equivalent

At the end of two years, there was an improvement in the mean preoperative sphere, cylinder, and spherical equivalent from −3.04 DS (±3.60), −3.63 DC (±1.82), and −4.70 D (±3.86) to −2.38 DS (±3.37), −2.80 DC (±1.48), and −3.75 D (±3.49), respectively. Statistically significant improvement (*P* < 0.001) was seen only in the cylindrical error.

### 3.3. Keratometry: Figure 1

There was a flattening of 2.04 D in the mean K1 and 2.07 D in mean K2 at the end of the 2-year follow-up, which was statistically significant (*P* < 0.001).

Three eyes of two patients with VKC and a history of eye rubbing showed progression at the end of two years. Hence, keratoconus patients with VKC had higher chances of failure of ACXL (3 out of 17 eyes with VKC, 17.65%) as compared to those without VKC who were stable two years after ACXL.

There were no complications noted after ACXL in any of the patients. Postoperatively, the mean time for epithelial healing was 3.32 ± 1.15 days. Mild haze was noticed in a majority of subjects on slit-lamp examination but did not have any effect on the visual acuity and subsided completely by eight weeks after surgery. The mean preoperative endothelial cell count was 2732.5 ± 174.08 cells/mm^3^ and showed no significant change at any of the postoperative visits up to two years (2689 ± 192.4; *P* = 0.36). There was also no alteration in the endothelial cell polymegathism (coefficient of variance; preoperative—36.12 ± 6.07; 2 years post-op—37.31 ± 6.12; *P* = 0.45) on specular microscopy. There was no evidence of delayed wound healing, ocular surface damage, or uveitis after ACXL in any of the patients.

## 4. Discussion

Corneal collagen cross-linking is an emerging treatment option for pediatric patients with keratoconus [8, 9]. The Siena CXL Pediatrics trial, the largest prospective study report involving 152 eyes of 77 patients (from 10 to 18 years) with the longest follow-up of 3 years demonstrated that, after CXL, keratoconus stabilized and demonstrated rapid and significant visual function improvement in pediatric patients [10]. They found an improvement in both UDVA and CDVA in patients under 18 years of age when compared to those in the 19–26 years age group, but it was not statistically significant. In patients over 27 years of age, there was a poorer functional response when compared with other age groups.

There have been various modifications to the original Dresden protocol when treating children [11]. It has been suggested that transepithelial CXL (epi-on) may be a better option in them as it is associated with lesser pain and provides a similar efficacy with fewer complications [12]. However, Malhotra et al. in their in vivo study on riboflavin penetration have shown that an intact epithelium blocks adequate penetration of riboflavin into the corneal stroma and hence reduces the effectiveness of CXL [13]. Additionally, a three-year follow-up study in corneas after epi-OFF or standard CXL compared with epi-ON CXL found better reduction in the steepest keratometry after the epi-OFF procedure [14]. There have also been changes in the protocols of the energy used and UV-A irradiation exposure time during CXL to shorten the duration of the procedure. Mrochen in his ex vivo experiments on “accelerated” cross-linking (AXCL) has shown that the biomechanical stiffening effect on corneal tissue using energies up to 10 mw/cm^2^ is similar to that with the standard protocol [15]. Cınar et al. compared conventional CXL with ACXL and found that at six months the change in UDVA and CDVA was statistically significant in the accelerated CXL group but did not reach statistical significance in the conventional CXL group [16].

Encouraged with the positive results of ACXL, we used it in the treatment of children less than 14 years of age with progressive keratoconus and found at the 2-year follow-up that there was a statistically significant improvement in the mean UDVA, CDVA, cylindrical refraction, and keratometry (K1 and K2). However, the improvement in the spherical refraction and SE was not statistically significant which may be explained by the small sample size of our study.

Vinciguerra et al. [17] demonstrated that the endothelial cell density did not alter after CXL in 40 eyes of 40 pediatric (from 9 to 18 years) after a two-year follow-up. In our study with ACXL, we found similar results of no significant reduction in the endothelial cell count at the end of two years. Failure of CXL to arrest progression of keratoconus is attributed to different genetic patterns, relative biomechanical modifications potentially occurring in the corneal stroma and the negative influences of other conditions such as allergy and atopy [18–21]. Ghosh et al. studied reviewed various proteomic and genomic expressions and discovered molecules that can serve as biomarkers which may have potential role in the management of keratoconus [20]. They also found that a history of allergy, atopy (eczema, asthma, and hayfever), corneal injury, eye rubbing, and rigid contact lens usage has been shown to be associated with the development of keratoconus. In our study we noticed a higher incidence of VKC in children with progressive keratoconus (55.5%). There was also a failure to stabilize keratoconus with ACXL in these patients (17.65%). Hence, management of the underlying cause in such children is of prime importance as persistent eye rubbing may “nullify” the effect of CXL. Topical treatment with steroids, mast cell stabilizers, and also the use of immunomodulators such as cyclosporine eye drops may help in alleviating allergy.

Other factors that may influence treatment are limbal stem cell (LSC) damage caused by chronic VKC [22] and UV-A damage [23]. The change in ocular flora due to chronic use of topical steroids in keratoconus patients with VKC can lead to an increased risk of postoperative keratitis [24]. Hence, while using ACXL to treat children, we recommend the usage of a limbal guard to protect the LSCs from damage and stoppage of the use of topical steroids (once the VKC is controlled) for at least two weeks prior to the procedure.

## 5. Conclusion

Very few studies have been published about the effectiveness of CXL in the younger age group. To the best of our knowledge this is the first study to evaluate the effectiveness of ACXL in children less than 14 years of age. The higher energy and shorter duration of treatment (9 mW/cm^2^ for 10 mins) of ACXL may prove to be a good option in children. It can potentially prevent amblyopia, improve the fit of contact lenses, and deter an early penetrating keratoplasty. For children with keratoconus and chronic VKC undergoing CXL, it would be prudent to treat the allergy aggressively with topical steroids during the active phase and with topical antiallergics and topical cyclosporine in chronic cases to reduce the chances of failure of CXL.

## Figures and Tables

**Figure 1:**
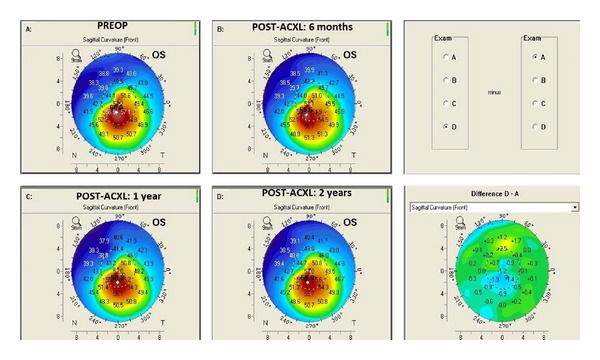
Pentacam difference map showing serial changes in sagittal curvature in an 11-year-old patient at preaccelerated corneal collagen cross-linking and postaccelerated corneal collagen cross-linking (6 months, 1 year, and 2 years).

**Table 1:** Table showing the mean UDVA (uncorrected distant visual acuity), CDVA (best corrected visual acuity), spherical and cylindrical refraction, SE (spherical equivalent), and K1/K2 (flat keratometry/steep keratometry) preoperatively and at 2-year postoperative period.

	Pre-op	2 years	*P* value
UDVA (in log⁡MAR)	0.76 (±0.26)	0.61 (±0.25)	0.005
CDVA (in log⁡MAR)	0.24 (±0.19)	0.12 (±0.12)	0.001
Spherical refraction (D)	−3.04 (±3.6)	−2.38 (±3.37)	0.28
Cylindrical refraction (D)	−3.63 (±1.82)	−2.80 (±1.48)	0.008
SE (D)	−4.7 (±3.86)	−3.75 (±3.49)	0.15
K1 (D)	48.53 (±3.57)	46.49 (±4.21)	0.001
K2 (D)	53.77 (±4.82)	51.70 (±5.41)	0.007
